# Low-Cost High-Throughput Genotyping for Diagnosing Familial Hypercholesterolemia

**DOI:** 10.1161/CIRCGEN.123.004103

**Published:** 2023-09-07

**Authors:** Shirin Ibrahim, Jeroen van Rooij, Annemieke J.M.H. Verkerk, Jard de Vries, Linda Zuurbier, Joep Defesche, Jorge Peter, Willemijn A.M. Schonck, Bahar Sedaghati-Khayat, G. Kees Hovingh, André G. Uitterlinden, Erik S.G. Stroes, Laurens F. Reeskamp

**Affiliations:** Department of Vascular Medicine (S.I., J.P., W.A.M.S., G.K.H., E.S.G.S., L.F.R.), Amsterdam UMC, University of Amsterdam, the Netherlands.; Department of Human Genetics (L.Z., J.D.), Amsterdam UMC, University of Amsterdam, the Netherlands.; Department of Experimental Vascular Medicine (J.P., G.K.H.), Amsterdam UMC, University of Amsterdam, the Netherlands.; Department of Internal Medicine, Erasmus MC, Rotterdam, the Netherlands (J.v.R., A.J.M.H.V., J.d.V., B.S.-K., A.G.U.).

**Keywords:** apolipoprotein B, genotyping, hypercholesterolemia, familial, LDL receptor, microarray analysis

## Abstract

**BACKGROUND:**

Familial hypercholesterolemia (FH) is a common but underdiagnosed genetic disorder characterized by high low-density lipoprotein cholesterol levels and premature cardiovascular disease. Current sequencing methods to diagnose FH are expensive and time-consuming. In this study, we evaluated the accuracy of a low-cost, high-throughput genotyping array for diagnosing FH.

**METHODS:**

An Illumina Global Screening Array was customized to include probes for 636 variants, previously classified as FH-causing variants. First, its theoretical coverage was assessed in all FH variant carriers diagnosed through next-generation sequencing between 2016 and 2022 in the Netherlands (n=1772). Next, the performance of the array was validated in another sample of FH variant carriers previously identified in the Dutch FH cascade screening program (n=1268).

**RESULTS:**

The theoretical coverage of the array for FH-causing variants was 91.3%. Validation of the array was assessed in a sample of 1268 carriers of whom 1015 carried a variant in *LDLR*, 250 in *APOB*, and 3 in *PCSK9*. The overall sensitivity was 94.7% and increased to 98.2% after excluding participants with variants not included in the array design. Copy number variation analysis yielded a 89.4% sensitivity. In 18 carriers, the array identified a total of 19 additional FH-causing variants. Subsequent DNA analysis confirmed 5 of the additionally identified variants, yielding a false-positive result in 16 subjects (1.3%).

**CONCLUSIONS:**

The FH genotyping array is a promising tool for genetically diagnosing FH at low costs and has the potential to greatly increase accessibility to genetic testing for FH. Continuous customization of the array will further improve its performance.

Familial hypercholesterolemia (FH) is a prevalent autosomal codominant disorder, predisposing affected individuals to premature atherosclerotic cardiovascular disease due to lifelong increased low-density lipoprotein cholesterol levels. FH variant carriers are at a 3- to 4-fold increased risk for atherosclerotic cardiovascular disease compared with noncarriers.^1–3^ Early initiation of lipid-lowering therapy in FH patients can prevent cardiovascular disease.^4^ Genetic screening is, therefore, strongly advised by guidelines, as it enables identification of FH patients at a young age, leads to a more reliable risk stratification, and positively affects initiation of and compliance to lipid-lowering therapy.^4–7^ Despite the widely acknowledged positive implications of an early and reliable genetic diagnosis, ≈90% of the 30 million affected individuals worldwide have not been identified yet, leading to significant losses in health in FH variant carriers.^6^ On top of several other promising strategies to enhance FH detection,^8^ improving access to cheap and reliable genetic testing for FH may help in further implementation of wider genetic testing to increase the diagnostic yield in subjects with a clinical suspicion of FH.

Nowadays, genetic testing for FH focuses on identifying a pathogenic variant in 1 of the 3 FH genes (ie, low-density lipoprotein receptor [*LDLR*], apolipoprotein B [*APOB*], and proprotein convertase subtilisin/kexin type 9 [*PCSK9*]). Genetic testing for FH has historically involved DNA sequencing of the 3 FH genes by Sanger sequencing in specialized laboratories, which is rapidly being replaced by next-generation sequencing (NGS) nowadays. Because such DNA sequencing methods are laborious and relatively expensive, large-scale application of DNA testing for FH is often hampered and alternative rapid and affordable molecular diagnostic methods are warranted.^9^ A promising method for fulfilling these demands is the use of genotyping arrays that have a standard content of predetermined FH-causing variants. While the ability of genotyping arrays to accurately diagnose very rare variants has been debated, multiple studies have demonstrated their diagnostic accuracy, particularly when modified genotype calling procedures are employed.^11–13^ For FH, this has been done with arrays containing probes for a limited amount of pathogenic variants, varying between 20 and 251 FH variants.^14–17^

In the present study, we first show that a more comprehensive customized genotyping array, containing 636 variants, previously classified as FH-causing, in combination with advanced genotyping calling procedures, would theoretically allow the identification of >90% of FH variant carriers in the Netherlands. Second, we validated this array in a selected sample of 1268 FH patients, previously diagnosed in the Dutch FH cascade screening program.

## METHODS

**Figure 1.**
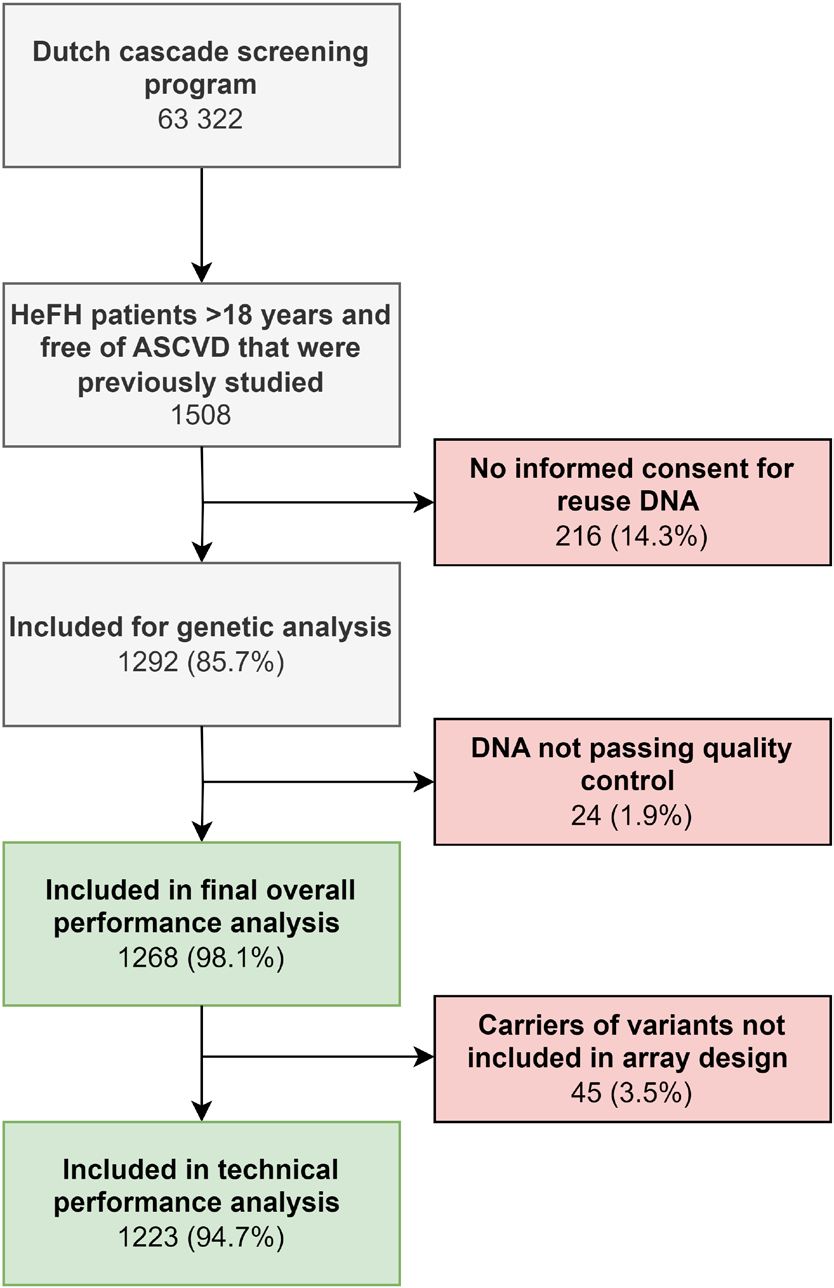
**Flowchart of participant inclusion in the current study.** The studied population was previously selected and studied for the effect of statins on cardiovascular disease (n=1508)^18^ and originated from the Dutch familial hypercholesterolemia (FH) cascade screening program, which included 63 322 participants. After QC, 1268 participants were included in the final overall performance analysis and 1223 in the technical performance analysis in the current study. ASCVD indicates atherosclerotic cardiovascular disease.

The methods of the current study are described in the Supplemental Material. In brief, an Illumina Global screening array was customized to include probes for 636 variants, previously classified as FH-causing variants. First, its theoretical coverage was assessed in all FH variant carriers diagnosed through NGS between 2016 and 2022 in the Netherlands (n=1772). Next, the performance of the array was validated in 1268 FH variant carriers previously identified in the Dutch FH cascade screening program (Figure 1). The reuse of anonymized data was approved by the Institutional Review Board of Amsterdam UMC (W20_033 20.061). Upon reasonable request, the data that support the findings of this study are available from the corresponding author.

## RESULTS

### Theoretical Diagnostic Yield

**Figure 2.**
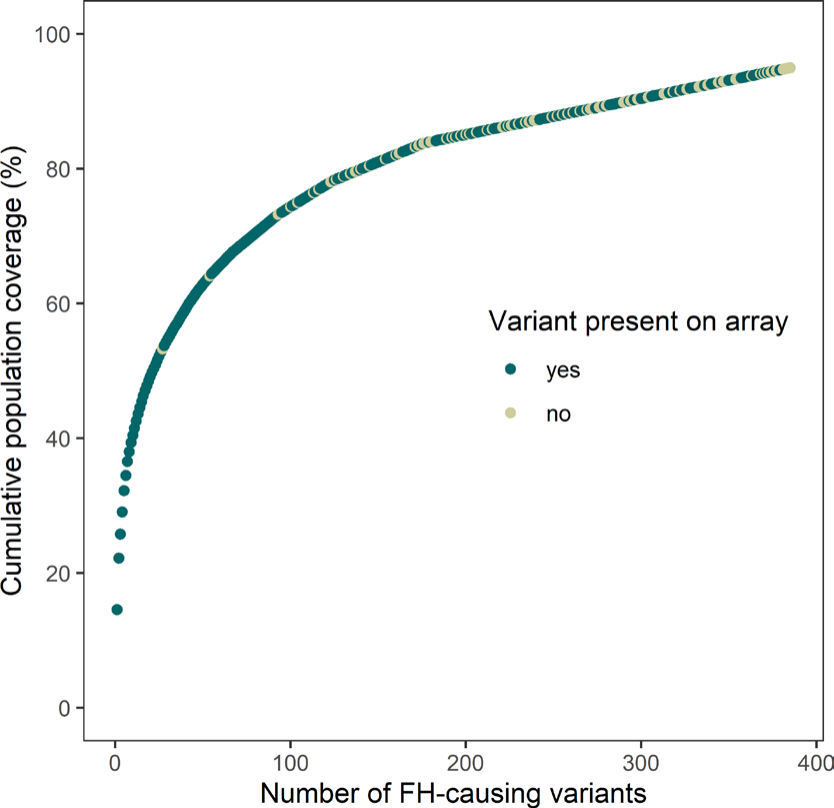
**Relationship between unique familial hypercholesterolemia (FH)-causing variants and cumulative population coverage.** Shown here is the cumulative proportion of FH cases in relation to the number of unique FH variants as diagnosed by next-generation sequencing in the Netherlands from June 2016 to June 2022. In dark green are those variants that are included in the FH genotyping array design, in light green those that are not.

To investigate the potential diagnostic yield of the customized FH genotyping array, its coverage of FH-causing single-nucleotide variants (SNVs) and insertions or deletions (indels) was compared with the detected FH-causing SNVs and indels in the Netherlands between July 2016 and July 2022. During these 6 years, 1772 FH variant carriers were identified, carrying a total of 385 unique variants (334 in *LDLR*, 18 in *APOB*, and 33 in *PCSK9*). Interestingly, 80% of FH cases in the Netherlands are caused by only 111 different variants (Figure 2). Theoretically, 1618 (91.3%) of these FH variant carriers should be detectable using the FH genotyping array as they carried an FH-causing variant that was included in the array design.

### Validation Cohort

A total of 1292 previously genetically diagnosed FH variant carriers were randomly selected from the Dutch national FH cascade screening program for reanalysis by the FH genotyping array. After quality control, 24 samples (carrying 9 unique variants) were excluded due to low DNA quality, resulting in a final cohort of 1268 FH variant carriers. The mean±SD age at start follow-up was 42.1±14.5 years, 52.7% was female. In this cohort, 1015 (80.0%) subjects carried a likely pathogenic or pathogenic variant in the *LDLR* gene, 250 (19.7%) in the *APOB* gene, and 3 (0.2%) in the *PCSK9* gene (Table 1). The analyzed cohort comprised 140 unique FH-causing variants of which 118 (84.3%) were SNVs, 16 (11.4%) were indels, and 6 (4.3%) were copy number variations (CNVs). Of these 140 unique variants, 122 (87.1%) were included in the design of the FH genotyping array and should thus be detectable. In total, 1223 (96.5%) of the subjects included in this study were carriers of an FH-causing variant that was included in the custom array design.

**Table 1.**
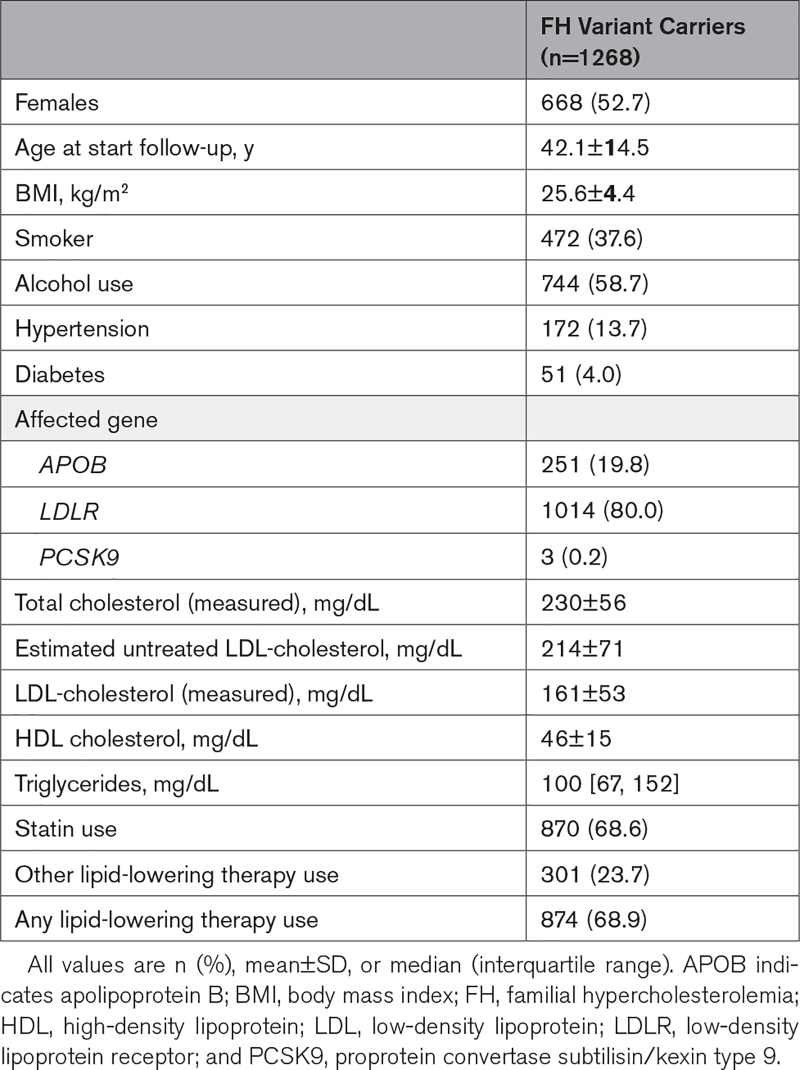
Characteristics of Studied FH Population

### Diagnostic Yield of FH Genotyping Array

First, the FH genotyping array performance was analyzed in the complete genotyped validation cohort, yielding the overall performance. Next, a technical performance was evaluated, which only included subjects in this cohort carrying variants included in the FH genotyping array design.

**Figure 3.**
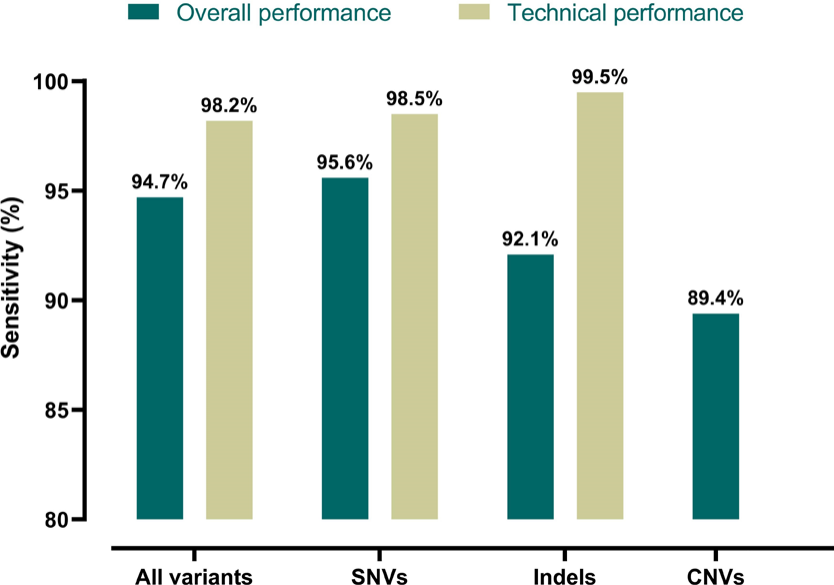
**Variant identification by familial hypercholesterolemia (FH) genotyping array.** FH genotyping array sensitivity in the complete cohort and according to variant type. Sensitivity was determined in the complete cohort (overall performance, dark green) and in those participants of which the variant was included as a probe in the genotyping array design (technical performance, light green). Because copy number variations (CNVs) are not directly included in the array design, but are detectable using array-based CNV analysis software, only the overall performance is reported. Indels indicates insertions or deletions; and SNVs, single-nucleotide variants.

Of the 1268 FH variant carriers included, 986 (77.8%) were carriers of an FH-causing SNV of which 957 (97.1%) were included in the FH genotyping array design. Of these SNV carriers, 943 were correctly identified by the array, yielding a sensitivity of 95.6% (943/986) in the overall performance analysis and a sensitivity of 98.5% (943/957) in the technical performance analysis (Figure 3). The study cohort also included 216 carriers of an FH-causing indel; 200 (92.6%) of which were included in the FH genotyping array design. Of the FH-causing indel carriers, 199 were correctly identified, resulting in a sensitivity of a 92.1% (199/216) in the overall performance analysis and a sensitivity of 99.5% (199/200) in the technical performance analysis (Figure 3). When categorized as detected variants per gene, 93.8% (952/1015) of *LDLR* variant carriers, 99.6% (249/250) of *APOB* variant carriers, and 0% (0/3) of *PCSK9* variant carriers were identified in the overall analyses. In the technical analysis, 98.0% (952/971) of *LDLR* variant carriers, 100% (249/249) of *APOB* variant carriers, and 0% (0/3) of *PCSK9* variant carriers were detected. In the final cohort, 66 carriers of a CNV in *LDLR* (59 deletions and 7 duplications) were included, of which 59 (55 deletions and 4 duplications) were correctly identified, resulting in a sensitivity of 89.4% (59/66; Figure 3).

The overall sensitivity of the FH genotyping array for SNVs, indels, and CNVs combined was 94.7% (Figure 3) with a false-negative rate of 5.3%. In 45 of the included carriers (3.5% of the total study cohort), the variants could not be investigated due to absence of variant-specific probes on the FH genotyping array. Excluding these subjects resulted in a 98.2% (1201/1223) sensitivity (Figure 3).

### Additionally Detected Variants

In 18 of the included subjects, 19 additional FH-causing variants (13 SNVs, 1 indel, and 5 CNVs) were identified (Table 2). These variants had not been detected previously using the gold standard sequencing methods. All 18 subjects were reanalyzed with targeted Sanger sequencing or multiplex ligation-dependent probe amplification for the newly identified variants. Seventeen of the 19 (89%) additionally identified variants (in 16 subjects) were absent upon resequencing and were considered a false-positive result. In the total cohort, this yields a false-positive rate of 1.3% (16/1268) and a specificity of 98.7%. Four of these 16 false positives were due to one probe specific for an FH-causing SNV in *LDLR* (Table 2). The remaining 2 SNVs were confirmed by targeted Sanger sequencing and were thus previously missed by the conventional diagnostic methods.

**Table 2.**
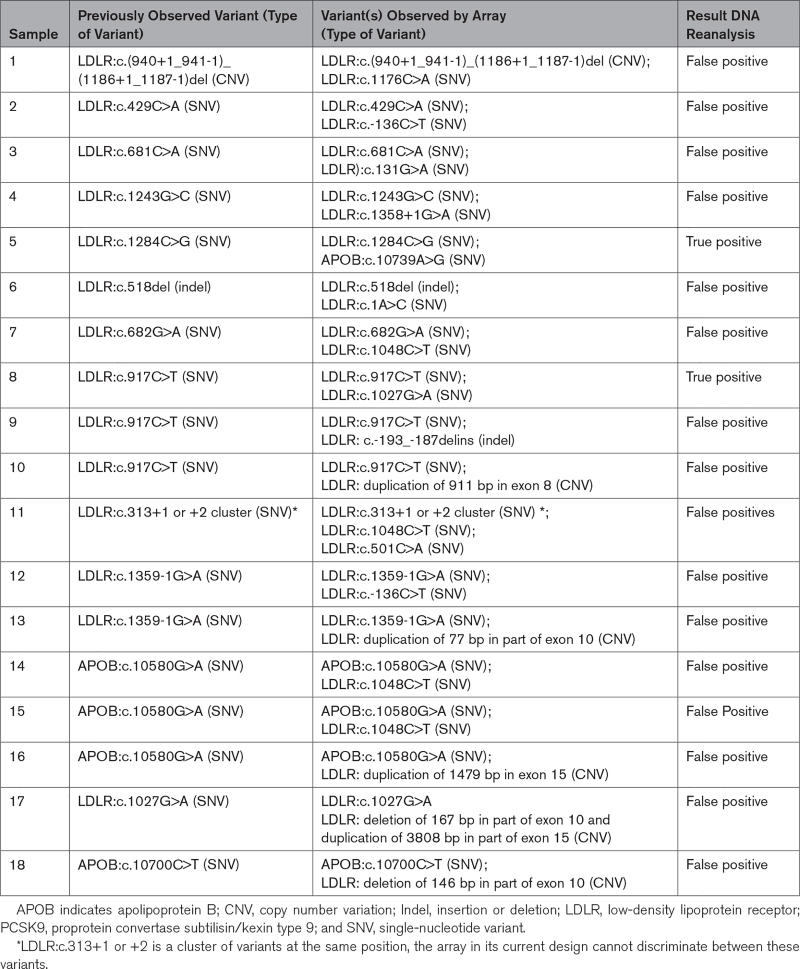
Additionally Identified Variants and Reanalysis Results

## DISCUSSION

The present study demonstrates that the use of a customized genotyping array, which included >600 FH-causing variants, holds promise as a high-throughput diagnostic modality for FH, with an observed overall sensitivity of 94.7% among 1268 previously diagnosed FH variant carriers. Due to many probes for variants in *LDLR* on this array, CNV analysis was also possible, although with a lower sensitivity of 89.4%. The overall specificity of the genotyping array was 98.2%.

The array was customized to include many FH-causing variant probes and performed well in a cohort of FH variant carriers. In contrast to the overall sensitivity in this cohort, which is especially relevant for clinicians and patients, the technical performance is of most interest for developers and suppliers. This study included 1223 carriers of an FH-causing variant that was detectable with the FH genotyping array, highlighting the importance of variant selection when designing genotyping arrays. Importantly, such arrays can easily be updated by suppliers and ongoing evaluation of newer array versions is expected to show an overall sensitivity that approaches its technical sensitivity. Furthermore, technical scrutiny of the false-positive variants could result in redesign of these probes and further improve the technical performance of the FH genotyping array as well.

The performance of uncustomized genotyping arrays in detecting rare pathogenic variants has been under debate.^10^ Here we show, however, that inclusion of rare variants—such as FH-causing variants—in the design of genotyping arrays, combined with advanced postprocessing algorithms, optimizes the array genotyping procedure and results in a high diagnostic yield.

The use of customized genotyping arrays for FH diagnosis is not a novel concept, but previous studies have evaluated only a limited number of FH-causing variants.^14^ Several genetic arrays for FH, which included common regional FH-causing variants in their design, have been described, including the Randox FH array evaluating 40 FH-causing variants, the Elucigene FH20 Array evaluating 20 FH-causing variants, and different versions of the LIPOchip evaluating 118 to 251 FH-causing variants.^15–17^ Although promising, implementation of these arrays has been hampered by their relatively limited number of variants, leading to significantly lower sensitivity compared with NGS.^14^ For example, one study showed that approximately two-third of all NGS-diagnosed FH variant carriers were missed by a limited-variant array containing 24 variants.^19^

For other diseases, such as amyotrophic lateral sclerosis and primary immunodeficiency, multiple studies have demonstrated successful use of arrays in detecting rare variants.^11–13^ The current study extends these optimistic findings to FH with the observation that inclusion of more variants leads to a greater diagnostic yield. The results regarding the overall sensitivity of the FH genotyping array are, however, not directly generalizable to other countries due to regional differences in FH-causing variants. With the current FH genotyping array design, theoretically, 91.3% of all FH cases in the Netherlands can be detected.

Approximately 10% of FH cases is caused by CNVs.^20^ CNV analysis with genotyping arrays is only possible when the probe density is sufficient. By virtue of the many included variants in *LDLR* in the FH genotyping array design, we were able to correctly detect 89.4% of CNVs, contrasting the majority of the previously reported FH-arrays, which were not designed to detect CNVs.^16^

The FH genotyping array does not solely include rare disease-causing variants, but also contains over 700 000 other variants, which enables the calculation of polygenic risk scores (PRS). In the case of FH, a PRS further enhancing coronary artery disease risk estimation may be highly relevant to tailor intensive treatment regimens to FH variant carriers at highest risk. A recent study showed that, compared with noncarriers with intermediate PRS, FH variant carriers with low PRS had only a 1.30-fold (95% CI, 0.39–4.32) increased coronary artery disease risk, while FH variant carriers in the highest quintile of the PRS distribution were characterized by a staggering 12.61 (95% CI, 2.96–53.62) increased coronary artery disease risk.^21^ Future research should focus on the clinical benefits of implementation of PRS reporting in FH.

### Future Promise of Genotyping Arrays in FH Detection and Diagnosis

In the present study, we were able to diagnose FH in ≈95% of the FH variant carriers at an array cost price of 30 Euros, using a technology that is more suitable for high-throughput FH screening at a population level due to high levels of automation, low turn-around time, and lack of secondary findings or variants of uncertain significance. The array cost price is only a fraction of the price of current DNA testing methods for FH in the Netherlands, which is over 2500 Euros, suggesting that use of the FH genotyping array might have a cost-saving effect.^22^ Nevertheless, the exact place of genotyping arrays in FH diagnostic workflows requires further debate. For example, because the FH genotyping array did not reach a 100% sensitivity, a clinical protocol should be in place to decide what should be done in terms of genetic analysis in cases with severe hypercholesterolemia and a negative array test result. The same holds true when using the FH genotyping array as a screening tool at a population level where the a priori probability of carrying an FH-causing variant is low. In both the settings—clinical testing and population screening—one could consider reanalysis using NGS in patients that are at high risk for carrying an FH-causing variant, for example those with high clinical probability according to the scores such as the Dutch Lipid Clinic Network criteria. In addition, to improve the false-positive rate of the array, a predefined period of simultaneous testing with the array and NGS could be considered. Validation studies, like the present one, are helpful to evaluate the areas of improvement in this regard. In combination with advanced processing algorithms, array genotyping procedures will further improve in calling rare variants. Importantly, the lack of a 100% sensitivity and specificity should be balanced against the low costs and effort required by array genotyping methods. Further evaluation of the possible application of microarray genotyping is warranted. However, considering the low costs, screening of larger cohorts of healthy individuals or those with subtle phenotypes (ie, mild hypercholesterolemia) seems sensible and realistic. Moreover, continuous customization of the array, for example by exclusion of probes that yield false-positive results and inclusion of additional probes for FH-causing variants from other geographic regions, can further improve its reliability and diagnostic yield, and can thus pave the way for cheaper worldwide screening of FH.

### Limitations

There are several limitations of the current study that warrant further discussion. First, the probes present on the current version of the array are based on the variants that were predominantly detected in the Netherlands and may therefore not be generalizable to all populations or ethnic groups. However, the array can easily be further customized to include more population-specific FH variants. Second, we selected a random population of FH variant carriers in the Netherlands, who only carried 140 unique variants, and thus, we were not able to test all probes included in the array design. Nevertheless, our study clearly shows the high overall performance of this array in a setting that resembles clinical practice in which an unselected population is genotyped, and it is likely that the performance for all included variants is similar to the currently observed results.

### Conclusions

Use of an affordable, customized FH genotyping array resulted in accurate identification of FH-causing variants at relatively low false-negative and false-positive rates in a cohort of previously diagnosed FH variant carriers. Continuous customization of the array will further increase the reliability of this technique and will allow for cheaper and more accessible genetic testing for FH.

## ARTICLE INFORMATION

### Acknowledgments

The authors thank all the participants of the Dutch familial hypercholesterolemia (FH) cascade screening program that gave consent for reuse of their genetic data for the current study.

### Sources of Funding

None.

### Disclosures

Dr Hovingh reports research grants from the Netherlands Organization for Scientific Research (vidi 016.156.445), CardioVascular Research Initiative, European Union and the Klinkerpad funds, institutional research support from Aegerion, Amgen, Astra-Zeneca, Eli Lilly, Genzyme, Ionis, Kowa, Pfizer, Regeneron, Roche, Sanofi, and The Medicines Company; speaker’s bureau and consulting fees from Amgen, Aegerion, Sanofi, and Regeneron until April 2019 (fees paid to the academic institution); and part-time employment and stock holder at Novo Nordisk A/S, Denmark since April 2019. Dr Stroes has received ad-board/lecturing fees, paid to the institution, from Amgen, Sanofi, Astra-Zeneca, Esperion, Daiichi-Sankyo, NovoNordisk, Ionis/Akcea, Amarin. Dr Reeskamp is cofounder of Lipid Tools and reports speakers’ fee from Ultragenyx. The other authors report no conflicts.

### Supplemental Material

Supplemental Methods

Supplemental Tables S1 and S2

References 18, 23–25

## Supplementary Material


