# Supplementary material for: Low-Cost High-Throughput Genotyping for Diagnosing Familial Hypercholesterolemia
Source: Circ Genom Precis Med. 2023 Sep 7;16(5):462–9. doi: 10.1161/CIRCGEN.123.004103 (PMC10581440; doi:10.1161/CIRCGEN.123.004103)
Supplement: Supplementary file 1 [file hcg-16-462-s001.pdf]

SUPPLEMENTAL MATERIAL

Low-cost high-throughput genotyping for diagnosing Familial Hypercholesterolemia

Content **Page**

Supplemental Methods 2

Supplemental Tables I-II 6

Methods

Sample selection

The present study included a cohort of heterozygous FH variant carriers, identified in the Dutch national FH cascade screening program.[18,23] This FH screening program, implemented between January 1994 and April 2013, led to genetic testing in over 60,000 individuals and the identification of more than 28,000 FH variant carriers through a “cascade screening” approach.[23] In this approach, first-degree relatives of newly identified FH index variant carriers were tested for the same variant. The cohort included in the current study, was previously selected in another study based on the linkage of health records to Pharmo Record Linkage System (RLS) to obtain clinical follow-up data.[18] In total, 1,292 FH variant carriers, aged 18 years or older, who provided informed consent for additional genetic analysis and were free of atherosclerotic cardiovascular disease at time of enrollment, were selected for further analysis in the present study (**Figure 1**). The reuse of anonymized data was approved by the Institutional Review Board of Amsterdam UMC (W20_033 # 20.061).

All carriers included in the present study were genetically diagnosed with FH through Sanger sequencing or multiplex ligation-dependent probe amplification (MLPA) of a specific FH-causing variant or CNV that was identified in another family member. Other FH-causing variants were not evaluated. All variants selected for analysis by the FH genotyping array in the present study are shown in **Supplementary Table I**.

Design FH genotyping array

The Illumina Global Screening Array version 3 MD (GSAv3MD-GOALLv1, hereinafter referred to as FH genotyping array, was custom designed in 2019 as part of the Genotyping On All Patients (GOALL) project at Erasmus MC in Rotterdam, the Netherlands, in an effort to include all FH-causing variants

identified at the national referral center for genetic dyslipidemias in the Netherlands during the last three decades. The initial number of known FH-causing single nucleotide variants (SNVs) and small insertions and deletions (indels) extracted from their database was 834. Due to technical limitations, a total of 557 SNVs and 79 indels were included in the final design of the array. Copy number variations (CNVs) were not directly included in the array design but are detectable using array-based CNV analysis software that uses CNV size and probe density of different genetic regions. Ultimately, the FH genotyping array in the present study was designed to detect 570 FH-causing variants in the *LDLR* gene, 43 in the *APOB* gene, and 23 in the *PCSK9* gene. These variants have previously been assessed by a panel of trained geneticists and were classified as pathogenic or likely pathogenic between 1994 and 2013 based on co-segregation of specific variants with a marked hypercholesterolemic phenotype within Dutch families that were included in the Dutch FH cascade screening program. Thus, classification of the variants included in the current version of the array design was not always performed according to current classification guidelines. A detailed description of the variants included in the design of the FH genotyping array is shown in **Supplementary Table II**.

Theoretical diagnostic yield

We first examined the array's theoretical coverage of FH-causing variants in the Netherlands. For this purpose, we selected all patients that were genetically diagnosed with FH at the national referral center for genetic dyslipidemias in the Netherlands between June 2016 and June 2022 and estimated the proportion of FH variant carriers that, in theory, should be identified using the final design of the FH genotyping array.

Genetic data analysis

Array Quality Control

Genotyping with the FH genotyping array was performed at the Human Genotyping Facility (HuGe-F) at Erasmus MC in Rotterdam, the Netherlands, after which the data was processed using Illumina GenomeStudio version 2.0 software. Subsequently, quality control (QC) analysis was performed using Plink version 1.9. For calling FH variants, a modified genotype calling protocol was applied. Variant QC was performed by removing variants that were not present in at least 95% of the subjects. Sample QC was performed by excluding individuals with a call-rate < 97.5% of all variants included in the array design. In addition, the zCall algorithm was applied to improve rare variant calling.[24] This algorithm is specifically designed for array-based technology and is implemented to improve variant calling as a post-processing step.[24]

CNV calling

GenomeStudio version 2.0 was used to generate input files for CNV calling. We used intra-batch clustering instead of predetermined cluster files to reduce noise in the input values for the CNV calling algorithms. CNV calling was first performed using PENNCNV, which is a software that automatically identifies CNVs through the incorporation of several parameters, such as total signal intensity (the Log(R) ratio), allelic intensity ratio at each SNP marker (the B-allele Frequency), the probe density, and the allele frequency of SNPs.[25] The PENNCNV output was filtered on 1) coverage of at least 10 probes and 2) a minimum PENNCNV confidence score of 20. In addition, any sample with a LogRdev > 0.18 (indicating inequality of the normal 2-allele intensity values) was excluded from further analyses. CNV calling was subsequently performed manually with NEXUS (Biodiscovery Research version 10) in subjects with a CNV according to the PENNCNV output. Nexus can be used to identify large chromosomal aberrations by making log-ratios and B-allele frequency plots in order to detect deletions, amplifications or polyploidy. CNVs were reported as duplications or deletions of one or more exons without exact nucleotide breakpoints positions. Any CNV that was found using

array data with PENNCNV and Nexus, but not previously diagnostically identified as the cause of FH in the specific individual were subsequently verified using MLPA.

Comparison FH genotyping array results with clinically ascertained pathogenic variants

Subjects included in this study were screened for all 636 FH-causing SNVs and indels included in the FH genotyping array design and underwent additional array-based CNV analysis. The dataset generated by this analysis was compared to the previously obtained sequencing data, which was considered the gold standard. Matched results from the FH genotyping array and Sanger sequencing or MLPA were considered as “true positives”. When the array result did not directly match with the previously found variant for a subject, the array results were considered as “false positive” or “false negative”. Samples with “false positive” results were reanalyzed using Sanger sequencing for SNVs and indels, and MLPA for CNVs, to rule out a previously missed variant. For all false negative results, it was investigated whether the previously found variant was included in the array design to evaluate whether a variant was missed by design or by technical performance of the array. Accordingly, two performance analyses were performed: 1) an overall analysis including all subjects in the study and 2) a technical analysis including only subjects carrying a variant which should have been detectable by the FH genotyping array.

Statistical analysis

Normally distributed data is expressed as mean $\pm$ SD, not normally distributed data as median [IQR]. Sensitivity was defined as the proportion of subjects that was correctly identified with the FH genotyping array as compared to previously performed sequencing. The specificity was defined as 1 minus the proportion of individuals with a false positive pathogenic variant identified by the FH genotyping array.

Supplementary Table I: FH-causing variants in study population

**Gene**	**FH-causing variant (n = 147)**	**Frequency (n)**
LDLR	LDLR:c.1690A>C	177
	LDLR:c.1359-1G>A	68
	LDLR:c.313+1G>C	61
	LDLR:c.917C>T	60
	LDLR:c.682G>A	57
	LDLR:2.500 base pair deletion of exon 7 and 8	49
	LDLR:c.1027G>A	47
	LDLR:c.621C>T	45
	LDLR:c.131G>A	39
	LDLR:c.241C>T	22
	LDLR:c.1285G>A	15
	LDLR:c.1898G>A	15
	LDLR:c.1775G>A	12
	LDLR:c.2054C>T	11
	LDLR:c.1057G>A	10
	LDLR:c.1358+1G>A	10
	LDLR:c.1048C>T	9
	LDLR:c.1307T>C	8
	LDLR:c.190+4A>G/T	8
	LDLR:c.2417insG	8
	LDLR:c.662A>G	8
	LDLR:c.801A>T	8
	LDLR:13.000 base pair deletion of promotor and exon 1	7
	LDLR:c.1004G>T	7
	LDLR:c.1284C>G	7
	LDLR:c.1783C>T	7
	LDLR:c.191-2A>G	7
	LDLR:c.429C>A	7
	LDLR:c.649G>A	7
	LDLR:c.681C>A	7
	LDLR:c.742T>G	7
	LDLR:4400 base pair insertion of exons 9 to 12 into intron 12	6
	LDLR:c.1247G>A	6
	LDLR:c.1291G>A	6
	LDLR:c.314-1G>A	6
	LDLR:c.763T>C	6
	LDLR:c.1265T>C	5
	LDLR:c.1329G>C	5
	LDLR:c.1898G>T	5
	LDLR:c.268G>A	5
	LDLR:c.-153C>T	4
	LDLR:c.1243G>C	4
	LDLR:c.1444G>A	4
	LDLR:c.1833G>C	4
	LDLR:c.1867_1868del	4
	LDLR:c.2191_2192ins13	4
	LDLR:c.2390-2A>G	4
	LDLR:c.394C>T	4
	LDLR:c.877del	4
	LDLR:c.-193_-187delinsTG	3
	LDLR:c.1118G>A	3
	LDLR:c.1195G>A	3
	LDLR:c.1340C>G	3
	LDLR:c.1601C>A	3
	LDLR:c.1678A>T	3
	LDLR:c.1759del	3
	LDLR:c.1784G>A	3
	LDLR:c.1820A>G	3
	LDLR:c.2389G>T	3
	LDLR:c.448C>T	3
	LDLR:c.648delTG	3
	LDLR:c.671A>T	3

	LDLR:c.858C>A	3
	LDLR:2000 base pair deletion of exon 16	2
	LDLR:c.1066G>T	2
	LDLR:c.1301C>G	2
	LDLR:c.1414G>T	2
	LDLR:c.1466A>G	2
	LDLR:c.1567G>A	2
	LDLR:c.1897C>T	2
	LDLR:c.28T>C	2
	LDLR:c.409G>A	2
	LDLR:c.518del	2
	LDLR:c.550T>C	2
	LDLR:c.660delC	2
	LDLR:c.798T>A	2
	LDLR:c.-134C>A	1
	LDLR:c.-136C>T	1
	LDLR:c.-139C>G	1
	LDLR:c.-172G>A	1
	LDLR:c.1056C>G	1
	LDLR:c.1176C>A	1
	LDLR:c.1255T>C	1
	LDLR:c.1297G>T	1
	LDLR:c.1324T>C	1
	LDLR:c.1432G>A	1
	LDLR:c.1516G>A	1
	LDLR:c.1519A>G	1
	LDLR:c.1586+5G>A	1
	LDLR:c.1586+5G>A/C	1
	LDLR:c.1603G>A	1
	LDLR:c.1769_1774del	1
	LDLR:c.1802A>G	1
	LDLR:c.1808A>G	1
	LDLR:c.1856T>C	1
	LDLR:c.188G>T	1
	LDLR:c.1916T>A	1
	LDLR:c.1988-1G>A	1
	LDLR:c.1A>C	1
	LDLR:c.1A>G	1
	LDLR:c.2001del	1
	LDLR:c.211G>C	1
	LDLR:c.2132G>A	1
	LDLR:c.2343_2347del	1
	LDLR:c.2476C>A	1
	LDLR:c.2478insC	1
	LDLR:c.263G>A	1
	LDLR:c.301G>A	1
	LDLR:c.313+1G>A	1
	LDLR:c.314-3C>T	1
	LDLR:c.463T>G	1
	LDLR:c.501C>A	1
	LDLR:c.519C>G	1
	LDLR:c.586C>A	1
	LDLR:c.643G>A	1
	LDLR:c.654_656del	1
	LDLR:c.661G>A	1
	LDLR:c.665G>T	1
	LDLR:c.682G>T	1
	LDLR:c.706T>C	1
	LDLR:c.828C>A	1
	LDLR:c.901G>T	1
	LDLR:c.915G>C	1
	LDLR:c.986G>A	1
	LDLR:deletion of exon 2 - exon 14	1
	LDLR:duplication of exon 7	1
APOB	APOB:c.10580G>A	161
	APOB:c.10580G>T	54
	APOB:c.10579C>T	15
	APOB:c.10672C>T	4

	APOB:c.10780T>C	4
	APOB:c.10700C>T	3
	APOB:c.10739A>G	3
	APOB:c.10508C>T	2
	APOB:c.10679A>G	1
	APOB:c.10708C>T	1
	APOB:c.13028_13029del	1
	APOB:c.13126C>T	1
	APOB:c.13184G>A	1
PCSK9	PCSK9:c.1405C>T	3

Supplementary Table II: Variants with specific probe included in FH genotyping array design

Gene	Type	Variants
LDLR	SNV	LDLR:c.1A>G, LDLR:c.-172G>A, LDLR:c.-153C>T, LDLR:c.-152C>T, LDLR:c.-151C>G, LDLR:c.-150A>G, LDLR:c.-139C>G, LDLR:c.131G>A, LDLR:c.-137C>T, LDLR:c.-136C>T, LDLR:c.-135C>G, LDLR:c.-134C>A, LDLR:c.-127G>C, LDLR:c.-121T>C, LDLR:c.-120C>T, LDLR:c.-79C>G, LDLR:c.-13A>G, LDLR:c.1A>C, LDLR:c.2T>C, LDLR:c.11G>A, LDLR:c.29G>A, LDLR:c.34G>A, LDLR:c.76A>T, LDLR:c.79T>C, LDLR:c.81C>A, LDLR:c.82G>T, LDLR:c.91G>A, LDLR:c.91G>T, LDLR:c.100T>G, LDLR:c.102C>G, LDLR:c.110G>A, LDLR:c.115T>C, LDLR:c.126C>A, LDLR:c.136T>G, LDLR:c.139G>A, LDLR:c.151G>A, LDLR:c.157C>T, LDLR:c.169G>A, LDLR:c.178C>T, LDLR:c.187T>G, LDLR:c.190+4A>T, LDLR:c.191-2A>G, LDLR:c.211G>C, LDLR:c.224G>A, LDLR:c.227G>A, LDLR:c.248T>C, LDLR:c.251C>T, LDLR:c.259T>G, LDLR:c.261G>A, LDLR:c.262A>G, LDLR:c.263G>A, LDLR:c.265T>A, LDLR:c.266G>A, LDLR:c.268G>A, LDLR:c.269A>G, LDLR:c.270T>A, LDLR:c.274C>G, LDLR:c.283T>C, LDLR:c.284G>T, LDLR:c.296C>G, LDLR:c.299A>C, LDLR:c.301G>A, LDLR:c.311G>A, LDLR:c.313+1G>A, LDLR:c.313+5G>T, LDLR:c.314-3C>T, LDLR:c.314-1G>A, LDLR:c.325T>C, LDLR:c.326G>A, LDLR:c.337G>T, LDLR:c.337G>A, LDLR:c.343C>T, LDLR:c.344G>A, LDLR:c.352G>T, LDLR:c.363C>G, LDLR:c.367T>C, LDLR:c.370C>G, LDLR:c.382T>C, LDLR:c.391G>C, LDLR:c.394C>T, LDLR:c.397G>T, LDLR:c.400T>C, LDLR:c.409G>A, LDLR:c.413C>G, LDLR:c.415G>A, LDLR:c.418G>A, LDLR:c.419A>G, LDLR:c.420G>C, LDLR:c.427T>G, LDLR:c.429C>A, LDLR:c.431C>T, LDLR:c.434T>C, LDLR:c.443G>A, LDLR:c.451G>C, LDLR:c.451G>A, LDLR:c.458T>G, LDLR:c.463T>C, LDLR:c.463T>G, LDLR:c.464G>T, LDLR:c.465C>A, LDLR:c.478T>C, LDLR:c.479G>A, LDLR:c.491T>G, LDLR:c.495G>A, LDLR:c.501C>A, LDLR:c.508G>A, LDLR:c.512C>T, LDLR:c.514G>A, LDLR:c.518G>A, LDLR:c.519C>A, LDLR:c.519C>G, LDLR:c.523G>A, LDLR:c.527G>T, LDLR:c.530C>T, LDLR:c.534T>G, LDLR:c.542C>T, LDLR:c.542C>G, LDLR:c.550T>C, LDLR:c.551G>A, LDLR:c.564C>G, LDLR:c.580A>G, LDLR:c.586C>A, LDLR:c.590G>T, LDLR:c.590G>A, LDLR:c.601G>A, LDLR:c.612C>A, LDLR:c.621C>T, LDLR:c.622G>A, LDLR:c.622G>T, LDLR:c.626G>A, LDLR:c.628A>C, LDLR:c.631C>T, LDLR:c.632A>T, LDLR:c.643C>T, LDLR:c.647G>A, LDLR:c.661G>A, LDLR:c.662A>G, LDLR:c.664T>C, LDLR:c.666C>A, LDLR:c.666C>T, LDLR:c.670G>A, LDLR:c.671A>T, LDLR:c.681C>A, LDLR:c.682G>C, LDLR:c.682G>T, LDLR:c.682G>A, LDLR:c.683A>G, LDLR:c.691T>G, LDLR:c.694G>A, LDLR:c.694+2T>C, LDLR:c.706T>C, LDLR:c.718G>A, LDLR:c.718G>T, LDLR:c.722T>C, LDLR:c.731C>G, LDLR:c.742T>G, LDLR:c.757C>T, LDLR:c.760C>T, LDLR:c.761A>C, LDLR:c.762G>T, LDLR:c.763T>G, LDLR:c.763T>A, LDLR:c.763T>C, LDLR:c.769C>T, LDLR:c.788A>G, LDLR:c.796G>C, LDLR:c.796G>A, LDLR:c.796G>T, LDLR:c.798T>A, LDLR:c.799G>A, LDLR:c.801A>T, LDLR:c.805G>C, LDLR:c.806G>A, LDLR:c.808T>A, LDLR:c.809G>A, LDLR:c.810C>A, LDLR:c.817+1G>A, LDLR:c.826T>G, LDLR:c.829G>A, LDLR:c.846C>A, LDLR:c.850T>G, LDLR:c.853C>T, LDLR:c.858C>A, LDLR:c.859G>A, LDLR:c.862G>A, LDLR:c.880A>G, LDLR:c.886T>C, LDLR:c.886T>A, LDLR:c.887G>A, LDLR:c.888C>A, LDLR:c.889A>C, LDLR:c.902A>G, LDLR:c.904T>G, LDLR:c.904T>C, LDLR:c.906C>G, LDLR:c.907C>T, LDLR:c.910G>A,, LDLR:c.911A>T, LDLR:c.915G>C, LDLR:c.917C>T, LDLR:c.919G>C, LDLR:c.922G>A, LDLR:c.938G>A, LDLR:c.939C>G, LDLR:c.939C>A ,LDLR:c.940G>T, LDLR:c.940+2T>G, LDLR:c.941-4G>A, LDLR:c.941-2A>G, LDLR:c.947A>G, LDLR:c.949G>A, LDLR:c.953G>A, LDLR:c.967G>A, LDLR:c.970G>T, LDLR:c.970G>A, LDLR:c.977C>G, LDLR:c.979C>T, LDLR:c.981C>A, LDLR:c.986G>A, LDLR:c.1004G>T, LDLR:c.1012T>G, LDLR:c.1024G>A, LDLR:c.1027G>A, LDLR:c.1033C>T, LDLR:c.1048C>T, LDLR:c.1054T>C, LDLR:c.1055G>A, LDLR:c.1057G>A, LDLR:c.1061-8T>C, LDLR:c.1061-1G>C, DLR:c.1061A>G, LDLR:c.1063A>G, LDLR:c.1065C>G, LDLR:c.1066G>T, LDLR:c.1069G>A, LDLR:c.1070A>G, LDLR:c.1073G>A, LDLR:c.1080T>G, LDLR:c.1085A>C, LDLR:c.1090T>C, LDLR:c.1102T>G, LDLR:c.1102T>C, LDLR:c.1103G>C, LDLR:c.1103G>A, LDLR:c.1118G>A, LDLR:c.1130G>A, LDLR:c.1132C>T, LDLR:c.1135T>C, LDLR:c.1136G>A, LDLR:c.1145G>T, LDLR:c.1151A>C, LDLR:c.1156G>T, LDLR:c.1166C>T, LDLR:c.1174T>A, LDLR:c.1176C>A, LDLR:c.1180G>A, LDLR:c.1186+5G>A, LDLR:c.1187-10G>A, LDLR:c.1187-7C>A, LDLR:c.1187-2A>G, LDLR:c.1189T>A, LDLR:c.1195G>A, LDLR:c.1196C>A, LDLR:c.1200C>A, LDLR:c.1201C>G, LDLR:c.1202T>A, LDLR:c.1207T>C, LDLR:c.1215C>G, LDLR:c.1216C>A, LDLR:c.1216C>T, LDLR:c.1217G>C, LDLR:c.1217G>A, LDLR:c.1222G>A, LDLR:c.1238C>T, LDLR:c.1241T>G, LDLR:c.1243G>C, LDLR:c.1244A>G, LDLR:c.1246C>T, LDLR:c.1247G>C, LDLR:c.1247G>T, LDLR:c.1247G>A, LDLR:c.1252G>A, LDLR:c.1252G>T, LDLR:c.1255T>C, LDLR:c.1265T>C, LDLR:c.1274A>T, LDLR:c.1284C>G, LDLR:c.1285G>A, LDLR:c.1291G>A, LDLR:c.1294C>G, LDLR:c.1297G>C, LDLR:c.1301C>G, LDLR:c.1301C>A, LDLR:c.1301C>T, LDLR:c.1307T>C, LDLR:c.1322T>A ,LDLR:c.1322T>C, LDLR:c.1324T>C, LDLR:c.1325A>G, LDLR:c.1328G>C, LDLR:c.1329G>C, LDLR:c.1330T>C, LDLR:c.1340C>G, LDLR:c.1342C>T, LDLR:c.1352T>C, LDLR:c.1358+1G>A, LDLR:c.1358+2T>A, LDLR:c.1359-2A>G, LDLR:c.1359-1G>A, LDLR:c.1361C>T, LDLR:c.1393T>A, LDLR:c.1394A>G, LDLR:c.1405A>T, LDLR:c.1406T>A, LDLR:c.1414G>T, LDLR:c.1415A>C, LDLR:c.1420C>T, LDLR:c.1426C>T, LDLR:c.1429G>A, LDLR:c.1432G>A, LDLR:c.1433G>A, LDLR:c.1435C>G, LDLR:c.1444G>A, LDLR:c.1447T>C, LDLR:c.1449G>T, LDLR:c.1454A>G, LDLR:c.1455C>A, LDLR:c.1463T>C, LDLR:c.1466A>G, LDLR:c.1467C>G, LDLR:c.1470G>A, LDLR:c.1474G>A, LDLR:c.1475A>G, LDLR:c.1495T>C, LDLR:c.1502C>T, LDLR:c.1504G>T, LDLR:c.1510A>G, LDLR:c.1516G>A, LDLR:c.1519A>G, LDLR:c.1520A>C, LDLR:c.1521G>C, LDLR:c.1525A>G, LDLR:c.1549T>C, LDLR:c.1558A>G, LDLR:c.1567G>A, LDLR:c.1574A>T, LDLR:c.1577C>G, LDLR:c.1586+1G>A, LDLR:c.1586+1G>T, LDLR:c.1586+2T>C, LDLR:c.1586+5G>A, LDLR:c.1594T>G, LDLR:c.1597T>A, LDLR:c.1599G>A, LDLR:c.1601C>A, LDLR:c.1603G>T, LDLR:c.1613C>A, LDLR:c.1618G>T, LDLR:c.1618G>A, LDLR:c.1633G>A, LDLR:c.1634G>A, LDLR:c.1637G>A, LDLR:c.1645G>A, LDLR:c.1646G>A, LDLR:c.1658A>G, LDLR:c.1678A>T, LDLR:c.1681C>T, LDLR:c.1684T>A, LDLR:c.1686G>A, LDLR:c.1688C>A, LDLR:c.1691A>C, LDLR:c.1691A>G, LDLR:c.1694G>T, LDLR:c.1702C>G, LDLR:c.1703T>C,

		LDLR:c.1705+1G>T, LDLR:c.1705+2T>C, LDLR:c.1706-10G>A, LDLR:c.1706-2A>C, LDLR:c.1706-1G>A, LDLR:c.1706A>T, LDLR:c.1715G>A, LDLR:c.1720C>T, LDLR:c.1721G>A, LDLR:c.1729T>C, LDLR:c.1729T>G, LDLR:c.1730G>C, LDLR:c.1743A>T, LDLR:c.1747C>T, LDLR:c.1761C>G, LDLR:c.1765G>A, LDLR:c.1774G>A, LDLR:c.1775G>A, LDLR:c.1783C>T, LDLR:c.1784G>A, LDLR:c.1784G>T, LDLR:c.1792A>C, LDLR:c.1793T>A, LDLR:c.1808A>G, LDLR:c.1816G>T, LDLR:c.1817C>A, LDLR:c.1820A>G, LDLR:c.1823C>T, LDLR:c.1825T>C, LDLR:c.1829C>G, LDLR:c.1833G>C, LDLR:c.1834G>T, LDLR:c.1835C>T, LDLR:c.1836C>A, LDLR:c.1836C>T, LDLR:c.1837G>A, LDLR:c.1843G>A, LDLR:c.1845+1G>A, LDLR:c.1845+2T>C, LDLR:c.1846G>A, LDLR:c.1853T>C, LDLR:c.1855T>C, LDLR:c.1856T>G, LDLR:c.1856T>C, LDLR:c.1864G>A, LDLR:c.1867A>G, LDLR:c.1868T>C, LDLR:c.1876G>A, LDLR:c.1879G>A, LDLR:c.1892C>A, LDLR:c.1897C>T, LDLR:c.1898G>A, LDLR:c.1898G>T, LDLR:c.1916T>A, LDLR:c.1937T>G, LDLR:c.1943C>T, LDLR:c.1946C>T, LDLR:c.1948G>A, LDLR:c.1954A>G, LDLR:c.1966C>A, LDLR:c.1976C>A, LDLR:c.1988-1G>A, LDLR:c.1998G>A, LDLR:c.1999T>C, LDLR:c.2000G>A, LDLR:c.2026G>C, LDLR:c.2026G>A, LDLR:c.2032C>T, LDLR:c.2039T>C, LDLR:c.2042G>A, LDLR:c.2043C>A, LDLR:c.2050G>A, LDLR:c.2054C>T, LDLR:c.2056C>T, LDLR:c.2059A>T, LDLR:c.2068C>G, LDLR:c.2089G>A, LDLR:c.2096C>T, LDLR:c.2101G>A, LDLR:c.2106G>A, LDLR:c.2113G>T, LDLR:c.2113G>C, LDLR:c.2119G>A, LDLR:c.2131T>A, LDLR:c.2140G>T, LDLR:c.2140+5G>A, LDLR:c.2140+103G>T, LDLR:c.2164C>T, LDLR:c.2177C>T, LDLR:c.2206G>A, LDLR:c.2230C>T, LDLR:c.2231G>A, LDLR:c.2242G>A, LDLR:c.2252G>A, LDLR:c.2282C>T, LDLR:c.2297C>T, LDLR:c.2308C>T, LDLR:c.2311+1G>T, LDLR:c.2312-3C>A, LDLR:c.2320G>T, LDLR:c.2324T>C, LDLR:c.2384C>G, LDLR:c.2384C>A, LDLR:c.2389G>T, LDLR:c.2389G>A, LDLR:c.2389+1G>T, LDLR:c.2389+1G>C, LDLR:c.2389+2T>A, LDLR:c.2390-2A>G, LDLR:c.2390-1G>C, LDLR:c.2411T>C, LDLR:c.2430G>A, LDLR:c.2431A>T, LDLR:c.2448G>C, LDLR:c.2475C>G, LDLR:c.2476C>A, LDLR:c.2479G>A, LDLR:c.2483A>G, LDLR:c.2506G>A, LDLR:c.2530G>A, LDLR:c.10580G>A
	Indel	LDLR:c.-187_-185del, LDLR:c.-138del, LDLR:c.9del, LDLR:c.62_65del, LDLR:c.227_233del, LDLR:c.230del, LDLR:c.303del, LDLR:c.313+4_313+16del , LDLR:c.320_332del, LDLR):c.340_344del, LDLR:c.347_349del, LDLR:c.378del, LDLR:c.457_465del, LDLR:c.467del, LDLR:c.478_486del, LDLR:c.513del, LDLR:c.518del, LDLR:c.532del, LDLR:c.563_569del, LDLR:c.565del, LDLR:c.645_646del, LDLR:c.648_649del, LDLR:c.654_656del, LDLR:c.654_656del, LDLR:c.669_676del, LDLR:c.680_681del, LDLR:c.702del, LDLR:c.784del, LDLR:c.796_799del, LDLR:c.820del, LDLR:c.877del, LDLR:c.925_931del, LDLR:c.948del, LDLR:c.962del, LDLR:c.1085del, LDLR:c.1112_1133del, LDLR:c.1187del, LDLR:c.1199_1207del, LDLR:c.1206_1207del, LDLR:c.1392del, LDLR:c.1478_1479del, LDLR:c.1486_1487del, LDLR:c.1658_1660del, LDLR:c.1683del, LDLR:c.1753del, LDLR:c.1759del, LDLR:c.1769_1774del, LDLR:c.1789del, LDLR:c.1842_1845+2del, LDLR:c.1853_1864del, LDLR:c.1867_1868del, LDLR:c.1871_1873del, LDLR:c.1886del, LDLR:c.1996_2012del, LDLR:c.2033_2044del, LDLR:c.2050_2063del, LDLR:c.2055_2067del, LDLR:c.2060del, LDLR:c.2063del, LDLR:c.2077_2078del, LDLR:c.2120_2140+3del, LDLR:c.2127_2129del, LDLR:c.2140+1_2140+12del, LDLR:c.2178del, LDLR:c.2191_2203dup, LDLR:c.2251del, LDLR:c.2274del, LDLR:c.2292del, LDLR:c.2343_2347del, LDLR:c.2400_2403del, LDLR:c.2402del, LDLR:c.2403_2406del, LDLR:c.2411del, LDLR:c.2416ins, LDLR:c.2500del
APOB	SNV	APOB:c.13288T>A, APOB:c.13220T>C, APOB:c.13200T>A, APOB:c.13196A>C,, APOB:c.13184G>A, APOB:c.13183G>A, APOB:c.13181T>C, APOB:c.13168G>C, APOB:c.13160A>T, APOB:c.13151T>C, APOB:c.13135C>T, APOB:c.13130T>C, APOB:c.13126C>T, APOB:c.13102C>G, APOB:c.13022T>A, APOB:c.13014A>G, APOB:c.12940A>G, APOB:c.11953G>A, APOB:c.10808A>G, APOB:c.10780T>C, APOB:c.10768G>A, APOB:c.10740C>G, APOB:c.10739A>G, APOB:c.10724G>A, APOB:c.10708C>T, APOB:c.10700C>T, APOB:c.10679A>G, APOB:c.10673G>A, APOB:c.10672C>T, APOB:c.10657G>A, APOB:c.10642G>A, APOB:c.10579C>T, APOB:c.10543A>G, APOB:c.10520G>C, APOB:c.10519C>T, APOB:c.10508C>T, APOB:c.10486A>G, APOB:c.10346A>C, APOB:c.10580G>T, APOB:c.13028_13029del
	Indel	APOB:c.13480_13482del, APOB:c.13320del,APOB:c.13242del
PCSK9	SNV	PCSK9:c.-11C>T, PCSK9:c.76G>A, PCSK9:c.137G>T, PCSK9:c.158C>G, PCSK9:c.169G>A, PCSK9:c.207+5G>A, PCSK9:c.212C>T, PCSK9:c.286C>T, PCSK9:c.311G>A, PCSK9:c.530G>A, PCSK9:c.644G>A, PCSK9:c.658G>A, PCSK9:c.1120G>T, PCSK9:c.1274A>G, PCSK9:c.1327G>A, PCSK9:c.1394C>T, PCSK9:c.1399C>G, PCSK9:c.1405C>T, PCSK9:c.1486C>T, PCSK9:c.1856A>C, PCSK9:c.2037C>A, PCSK9:c.2038C>T
